# DETECTION OF AI-GENERATED IMAGES: A MIXED METHODS STUDY ON AGE-RELATED DIFFERENCES

**DOI:** 10.1093/geroni/igae098.4158

**Published:** 2024-12-31

**Authors:** Enilda Velazquez, Gabriela Flores-Cruz, Nelson Roque

**Affiliations:** University of Central Florida, Orlando, Florida, United States; University of Central Florida, Orlando, Florida, United States; Pennsylvania State University, University Park, Pennsylvania, United States

## Abstract

Generative artificial intelligence (AI) content has become widely accessible but continues to face misuse for spreading misinformation that often targets older adults. This is concerning considering that AI generated content is projected to occupy 10% of the internet by 2025 (Gartner, 2024). Previous research has implicated age and analytical thinking as potential predictors in how well individuals identify misinformation (Pehlivanoglu et al., 2022; see CISDA model, Frazier et al., 2019). This study uses a mixed methods approach to examine the vulnerability of individuals across age groups (young adulthood: 18-39, middle adulthood: 40-59, old adulthood: 60-89; within-subjects) to discern AI manipulated images (AI-generated, non-AI generated; between-subjects) by using (1) signal detection analyses (i.e., hit, miss, false alarm, correct rejection) and (2) response time measures while controlling for analytical thinking (Cognitive Reflection Test score, Frederick, 2005) as an external variable (N = 190). A mixed methods ANOVA suggests that older adults (M = 0.426, SD = 0.150) exhibited significantly higher false alarms than younger adults (M = 0.306, SD = 0.180, p = 0.007, n2p = 0.053). However, there were no differences of age cohort on the rate of hits, misses, or correct rejections, nor on image discernment reaction time while controlling for analytical thinking. The increased false alarm rate among older adults may reflect AI sensitivity toward images, potentially indicating that AI sensitivity acts as a protective factor in the AI interactions of older adult’s AI interactions. Future research should explore this sensitivity as a safeguard against misinformation exploitation.

